# Addressing Selection and Confounding Biases in Dental Claims Data: A Causal Inference Framework for Periodontal–Systemic Disease Research

**DOI:** 10.1177/00220345251387660

**Published:** 2025-11-25

**Authors:** J.J. Wong, O. Urquhart, A. Carrasco-Labra, E.F. Schisterman, M. Glick

**Affiliations:** 1 Department of Biostatistics, Epidemiology, and Informatics, Perelman School of Medicine, University of Pennsylvania, Philadelphia, PA, USA; 2 Center for Integrative Global Oral Health School of Dental Medicine, University of Pennsylvania, Philadelphia, PA, USA

**Keywords:** oral disease, systemic disease, epidemiology, big data, dental public health, disease

## Abstract

Administrative health care data offer unique opportunities to investigate relationships between oral and systemic diseases. However, these data sources introduce methodological challenges that can compromise causal inference. This article demonstrates how, in the context of claims databases, selection bias (i.e., arising from restricting analyses to individuals with both dental and medical insurance) creates a collider structure that can distort estimates of periodontal treatment effects on systemic disease outcomes. Drawing on causal inference theory, we distinguish between confounding (resulting from common causes) and selection bias (resulting from common effects) and demonstrate how directed acyclic graphs (DAGs) can identify these biases and inform rigorous analytical strategies. Therefore, the goal of this article is to demonstrate how selection and confounding biases in administrative health care claims data can compromise causal inference in periodontal–systemic disease research and to introduce methodological approaches for addressing these threats. Our review of 7 studies investigating periodontal–systemic disease associations using claims data reveals methodological gaps in addressing selection bias in the current literature. Moreover, through a numerical example, we illustrate how selection bias can not only distort but also potentially reverse observed associations, producing contradictory clinical recommendations. To address these methodological threats, we introduce established causal inference strategies, referencing implementation tutorials: for confounding, we reference G-methods (G-formula, inverse probability weighting) and stratification-based approaches (regression, matching); for selection bias, we reference inverse probability of selection weighting approaches when data on nonselected individuals are available. To improve methodological rigor in oral–systemic research, we advocate for (1) routine use of DAGs with freely available software, (2) application of bias-correction techniques using established statistical packages, and (3) transparent reporting of bias assessment procedures. Strengthening causal inference methodology in dental research is paramount to building a robust evidence base on periodontal–systemic relationships that supports clinical decision making and integration of oral health into broader health care frameworks.

## Introduction

In this era of “big data” derived from administrative health care sources, advancing our understanding of oral and systemic disease relationships through observational research has become increasingly feasible (Kruse et al. 2016). Large-scale administrative databases enable investigators to study populations exceeding 100,000 participants (sample sizes unattainable through randomized controlled trials) while capturing real-world treatment patterns, long-term outcomes, and diverse patient populations with multiple comorbidities that are often excluded from traditional clinical trials, providing essential information on safety, effectiveness, and economic performance that complements findings from randomized controlled trials (Blonde et al. 2018). Realizing the full potential of administrative data requires implementing methodologically rigorous study designs and advanced analytic strategies to ensure valid causal inference. While these large datasets offer valuable real-world evidence, studies leveraging these resources are inherently susceptible to selection and confounding biases (Kruse et al. 2016; Nørgaard et al. 2017; Dahlen and Charu 2023). Dental investigators must be particularly vigilant in addressing these methodological limitations, as uncorrected biases may not only undermine causal inference but also create misleading conclusions and potentially compromise both evidence-based dental practice and patient outcomes (Prada-Ramallal et al. 2019).

**Figure 1.**
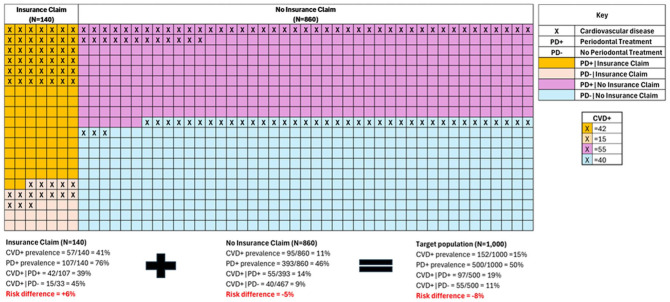
Illustration of a selection bias numerical example: observed associations between periodontal treatment and cardiovascular disease in insurance claims data versus the target population. This figure demonstrates how selection bias can reverse causal conclusions when analyzing periodontal–systemic relationships using insurance claims data. The visualization presents 3 populations: those with insurance claims (*n* = 140, left grid), those without insurance claims (*n* = 860, right grid), and the combined target population (*n* = 1,000, bottom calculations). Color coding distinguishes key subgroups: yellow cells represent individuals with cardiovascular disease (CVD) who received PT (CVD+|PT+), light purple cells represent individuals with CVD who did not receive PT (CVD+|PT−), light blue cells represent individuals without CVD who received PT, and white cells represent individuals without CVD who did not receive PT. In the insurance claims population, CVD prevalence is 39% (42/107) among PT-treated individuals versus 45% (15/33) among untreated individuals, suggesting a protective effect of PT with a risk difference of +6%. In contrast, the noninsurance population shows a CVD prevalence of 14% (55/393) among PT-treated individuals versus 9% (40/467) among untreated individuals, suggesting a harmful effect with a risk difference of −5%. When examining the total target population, the true causal relationship becomes clear: the CVD prevalence is 19% (97/500) among PT-treated individuals versus 11% (55/500) among untreated individuals, yielding a risk difference of −8%, indicating that PT is associated with increased CVD risk. This example illustrates how conditioning on insurance status (a collider) creates divergent and misleading associations in different subpopulations, potentially leading researchers to draw fundamentally opposite conclusions about causal relationships depending on which population is studied.

Insurance claims databases represent the most widely used yet methodologically challenging administrative data source when investigating effects of periodontal treatment (PT) on systemic disease outcomes (Fig. 1, Box). The primary limitation stems from inherent restriction to individuals with dual coverage requirements: dental insurance (required for documenting PT through CDT procedure codes) and medical insurance (required for documenting systemic conditions through ICD diagnosis codes). This dual coverage requirement creates what epidemiologists term a “collider structure,” introducing selection bias that can systematically distort causal estimates (Hyman 2015; Holmberg and Andersen 2022). Compounding this challenge, confounding bias emerges when common causes of both treatment assignment and outcomes generate structural associations unrelated to the causal effect of interest (Nørgaard et al. 2017). Although selection and confounding biases represent distinct threats to internal validity, they frequently co-occur and interact, creating overlapping biases through both selection and confounding mechanisms that can alter observed relationships between periodontal interventions and systemic health outcomes if left unaddressed (Hernán et al. 2004; Schneeweiss and Avorn 2005).

**Box.** Example of Selection Bias Reversing Causal Effects in Claims Analysis.

To illustrate the magnitude and clinical significance of selection bias in dental–systemic disease research, consider Figure 1, which presents a hypothetical numerical example of how selection bias can distort causal conclusions when using claims data. This example examines the association between periodontal treatment and cardiovascular disease (CVD) across 3 populations: (1) those with claims coverage (*n* = 140), (2) those without claims coverage (*n* = 860), and (3) the total target population (*n* = 1,000).
In the claims coverage population (*n* = 140), we observe that among 107 individuals who received periodontal treatment (PT+), 42 had CVD (39% CVD risk). Among 33 individuals who did not receive periodontal treatment (PT−), 15 had CVD (45% CVD risk). The risk difference of +6% suggests that not having PT increases CVD risk.
In stark contrast, among those without claims coverage (n = 860), among 393 individuals receiving periodontal treatment (PT+), 55 had CVD (14% CVD risk), while among 467 individuals not receiving periodontal treatment (PT−), 40 had CVD (9% CVD risk). The risk difference of −5% suggests the opposite effect.
Most critically, when examining the total target population (*n* = 1,000), among 500 individuals receiving PT (PT+), 97 had CVD (19% CVD risk), while among 500 individuals not receiving periodontal treatment (PT−), 55 had CVD (11% CVD risk). The true causal risk difference in the target population is −8%, indicating that not having PT reduces CVD risk. This reversal would lead to dramatically different clinical recommendations depending on which population is studied, demonstrating that selection bias is not merely a theoretical concern but a practical threat to the validity of dental–systemic disease research that can completely reverse the understanding of important causal relationships. When external data are available on uninsured populations, the inverse probability of selection weighting or transportability methods could be used to estimate effects in the broader target population, although such external data are rarely available in dental research settings.

Advances in causal inference methodology, particularly the development of g-methods and target trial emulation frameworks, provide robust solutions to these challenges (Naimi et al. 2017; Hernan and Robins 2020). Our systematic review of 7 recent studies examining periodontal–systemic relationships using claims data reveals systematic patterns of inadequately addressed selection and confounding biases inherent in claims-based analyses (Appendix Table) (Raedel et al. 2019; Choi et al. 2021; Blaschke et al. 2022; Beukers et al. 2023; Michalowicz et al. 2023; Thakkar-Samtani et al. 2023; Sakamoto et al. 2025). Accordingly, the aims of this article are to (1) demonstrate how selection and confounding biases in administrative health care claims data can compromise causal inference, (2) distinguish between these bias mechanisms using causal inference theory, and (3) introduce established methodological approaches and refer readers to implementation resources for enhancing the methodological rigor of future observational studies in dental research.

## The Counterfactual Framework for Causal Inference

The potential outcomes framework represents a foundational paradigm shift in modern epidemiology, formalizing rigorous causal reasoning that moves beyond simple associational thinking. This framework provides a principled foundation for understanding when and how we can draw causal conclusions from observational data, establishing clear assumptions necessary for valid causal inference. Recent advances in dental epidemiology have begun incorporating these methods, with studies using marginal structural models and inverse probability weighting to assess causal relationships in oral health outcomes (Peres et al. 2023).

Using causal inference, dental researchers can frame oral–systemic study questions using the potential outcomes framework, which addresses: *what would happen to each individual if they received treatment versus if they did not* (Rubin 2005)*?* This framework defines potential outcomes $Y^{a}$ , denoted as $Y_{i}^{a\;=\;1}$ if an individual i receives treatment (A=1) and $Y_{i}^{a\;=\;0}$ if they do not (A=0) , representing outcomes that could occur under each treatment condition (Hernán and Robins 2016). These potential outcomes (often termed “counterfactuals”) are central to causal analysis because they allow investigators to conceptualize treatment effects by comparing observed outcomes with what would have occurred under alternative clinical scenarios.

Although these potential outcomes can be conceptually defined for every individual, only 1 outcome is ever directly observable: the outcome corresponding to the treatment actually received. This limitation is known as the “fundamental problem of causal inference,” meaning individual causal effects $\left(Y_{i}^{a\;=\;1}-Y_{i}^{a\;=\;0}\right)$ cannot be directly measured (Höfler 2005). Instead, dental investigators must estimate causal effects at the population level by comparing mean potential outcomes under treatment and control, expressed as $E[Y^{a\;=\;1}]$ and $E\left[Y^{a\;=\;0}\right]$ , respectively (Elwert and Winship 2002). The average causal effect (ACE), defined as *$E\left[Y^{a\;=\;1}\right]-E[Y^{a\;=\;0}]$ *), represents the expected difference in glycemic control if all patients received periodontal therapy compared with if none of them did.

To validly estimate this ACE from observational claims data, 3 causal inference assumptions must be satisfied. First, “consistency” requires that treatment be well-defined and manipulable, ensuring that if an individual receives specific PT, the observed outcome must match the potential outcome for that treatment. This assumption is more nuanced than it initially appears, as it requires that the treatment under study be a well-defined, manipulable intervention, even if hypothetical. For instance, what constitutes “periodontal therapy” must be precisely defined rather than representing a heterogeneous mix of scaling, root planing, and surgical procedures with varying protocols. The consistency assumption demands that all variations of treatment coded under the same procedure code represent sufficiently similar interventions for causal inference purposes (Cole and Frangakis 2009).

Second, “positivity” ensures that every relevant covariate pattern has a nonzero probability of receiving each treatment level (Petersen et al. 2012). This assumption requires that there are both treated and untreated individuals within all strata defined by confounders and prior exposure levels. Positivity can be violated through structural zeros (when certain individuals cannot possibly receive treatment due to clinical contraindications) or random zeros (when chance or small sample sizes result in empty cells). For example, in periodontal research, positivity would be violated if all patients with severe diabetes automatically received intensive periodontal therapy, leaving no untreated comparison group within that stratum. Violations can be assessed by examining the distribution of propensity scores: extremely low or high probabilities of treatment indicate potential positivity problems that may require restriction of the inference to strata with adequate overlap or smoothing under parametric assumptions.

Finally, “exchangeability” guarantees that treated and untreated periodontal patient groups are comparable in terms of both measured and unmeasured confounders (Lindley and Novick 1981). This article emphasizes the “exchangeability” assumption and examines how its violation through confounding bias and selection mechanisms in insurance claims data can compromise the validity of periodontal–systemic disease research.

## Confounding through Common Causes

In randomized controlled trials, exchangeability is guaranteed by random allocation of PT, which balances both measured and unmeasured confounders between groups at baseline (Sibbald and Roland 1998). In contrast, observational studies using administrative data cannot leverage randomization. Instead, PT assignment is influenced by factors such as socioeconomic status, oral health literacy, or health-seeking behaviors. These variables create confounding when they serve as common causes of both the periodontal exposure A and outcome Y (Pourhoseingholi et al. 2012).

To determine whether covariates are sufficient for achieving conditional exchangeability in observational studies, investigators can draw directed acyclic graphs (DAGs): visual tools in which variables are represented as nodes and causal relationships as directed arrows (Tennant et al. 2021). DAGs help distinguish between direct causal paths of interest (e.g., A→Y) and backdoor paths that introduce bias. A backdoor path represents a noncausal association between exposure A and outcome Y arising when a common cause (confounder L) creates an association unrelated to the causal effect of interest, following the pattern A←L→Y (Maathuis and Colombo 2015).

**Figure 2.**
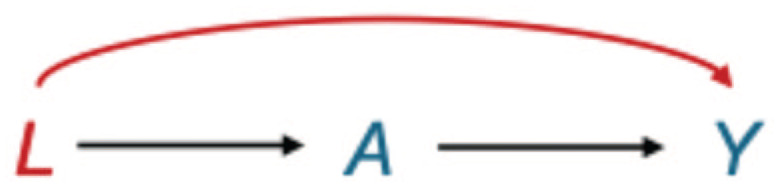
The classic confounding structure: smoking (*L*) as a common cause of periodontal disease severity (*A*) and diabetes risk (*Y*). This directed acyclic graph illustrates the classic structure of confounding bias, where smoking (*L*) acts as a common cause of both periodontal disease severity (*A*) and diabetes risk (*Y*), creating a backdoor path ( A←L→Y ). The observed association between periodontal disease severity and diabetes is influenced by 2 distinct causal pathways: (1) the direct causal path ( A→Y ), which represents the effect we aim to estimate, and (2) the backdoor path A→L→Y , which arises when smoking is not adjusted for. The red arrow from smoking to diabetes ( L→Y ) represents the confounding pathway that creates a noncausal association between *A* and *Y* through their common cause of smoking. This structure violates the exchangeability assumption, which requires that the distribution of potential outcomes *Y*^a^ is independent of exposure *A* given confounders *L* (i.e., $Y^{a}∐A|L$ ). Without conditioning on smoking, the crude association between periodontal disease and diabetes will be biased, as it captures both the causal effect and the noncausal association through the backdoor path. By adjusting for smoking status, the backdoor path will be blocked and conditional exchangeability can be achieved to ensure that the treated and untreated groups are comparable within levels of smoking.

Figure 2 illustrates this concept considering the relationship between periodontal treatment (A) and diabetes risk (Y), with smoking (L) acting as a confounder. Smoking is a common cause of both periodontal disease severity and diabetes risk, creating a backdoor path that biases the observed association (Martinez-Canut et al. 1995; Willi et al. 2007). To isolate the direct causal effect of periodontal treatment on diabetes risk, researchers must condition on smoking status to block this backdoor path (Maathuis and Colombo 2015). The backdoor criterion provides formal requirements for confounding adjustment: covariates L must block all backdoor paths between A and Y while containing no descendants of A (VanderWeele and Robins 2007).

Adjusting for measured confounders in periodontal–systemic research includes 2 main analytical technique families: G-methods (G-formula, inverse probability weighting, and G-estimation) and stratification-based approaches including regression, restriction, and matching (Naimi et al. 2017). When implementing these methods, investigators must apply subject matter knowledge to identify minimally sufficient covariate sets for confounding control while carefully avoiding adjustment for variables affected by both treatment and outcome, as such adjustments may introduce selection bias rather than reduce confounding bias.

## Selection Bias through Common Effects

Unlike confounding, which occurs when an unconditioned common cause of A and Y creates a backdoor path (requiring adjustment to block this path), selection bias occurs when conditioning on a common effect of A and Y creates a backdoor path (requiring that we avoid adjusting for this variable to prevent opening the path) (Hernán et al. 2004). Selection bias represents another potential source of nonexchangeability between treated and untreated periodontal patients (Hernán and Monge 2023).

**Figure 3.**
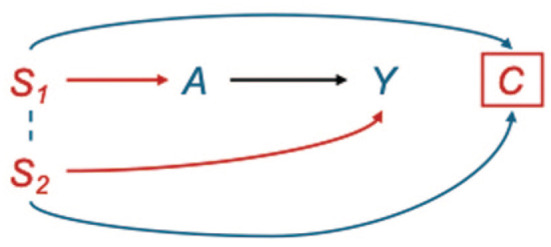
Selection bias in claims data: dental and medical insurance status as a common effect of periodontal disease severity (*A*) and diabetes risk (*Y*). This directed acyclic graph illustrates the collider structure that arises when studying the causal relationship between periodontal disease severity (*A*) and diabetes risk (*Y*) using claims data. Inclusion in the database (*C*) requires both dental insurance (*S_1_*), required for documenting periodontal treatment through CDT procedure codes, and medical insurance (*S_2_*), required for documenting diabetes through ICD codes. As a result, “being present in the database” (*C*) acts as a common effect of *A* and *Y* via *S_1_* and *S_2_*, forming a collider structure (*A*→ *C* ← *Y*). Conditioning on *C* inherently occurs when using claims data, creating a noncausal association between the exposure and outcome through this collider path. The dotted line between *S*_1_ (dental insurance status) and *S*_2_ (medical insurance status) represents their correlation, as patients with dental insurance are generally more likely to have medical insurance. This correlation further amplifies the spurious association between periodontal disease and diabetes when selection bias is not addressed. Unlike confounding bias, which can be resolved by conditioning on common causes, selection bias emerges because of conditioning on a common effect, making it particularly challenging in claims-based research, where the data structure itself implies this conditioning. Without accounting for this selection mechanism, effect estimates will be biased away from the true causal effect, potentially leading to incorrect conclusions about the periodontal–diabetes relationship.

Selection bias occurs when, in an attempt to identify a causal effect, we condition on a variable that is a common effect (a “collider”) of 2 other variables, one of which is either the exposure or a cause of the exposure and the other is either the outcome or a cause of the outcome. In DAG terminology, a collider is represented as a node in which 2 arrows converge (A→C←Y). Figure 3 illustrates this collider structure in studying the causal relationship between periodontal treatment (A) and diabetes risk (Y) using insurance claims data. Inclusion in the database (C) requires both dental coverage (S_1_), necessary for documenting periodontal treatment through CDT procedure codes, and medical coverage (S_2_), required for recording diabetes diagnoses through ICD codes. Consequently, “being present in the database” (C) acts as a common effect of A and Y via S_1_ and S_2_, forming a collider structure.

When C is conditioned on (by including only individuals who appear in claims databases), researchers inadvertently open a backdoor path between A and Y via S_1_ and S_2_ (Elwert and Winship 2014). Unlike confounding bias (in which adjustment for covariates blocks backdoor paths), selection bias requires caution because conditioning on a collider creates new noncausal pathways.

**Figure 4.**
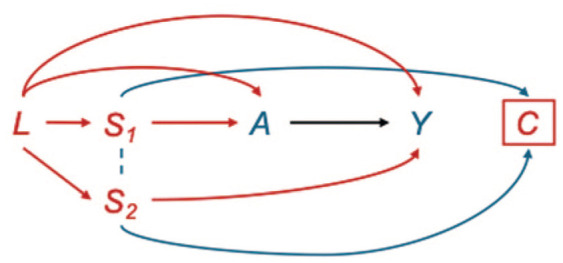
Compounded selection and confounding bias structures: socioeconomic factors (L) as common causes of both selection mechanisms and health outcomes in claims-based research. This directed acyclic graph illustrates the complex bias structure that arises when socioeconomic factors (*L*) are common causes of both insurance status selection mechanisms and health outcomes of interest. The measured common cause *L* creates multiple confounding pathways (*L* → *A* → *Y* and *L* → *Y*) that require adjustment to estimate the causal effect of periodontal disease severity (*A*) on diabetes risk (*Y*). In addition, *L* influences dental insurance status (*S*_1_) and medical insurance status (*S*_2_), which jointly determine inclusion in the claims database (*C*). The blue arrows from *S*_1_ and *S*_2_ to *C* represent this selection mechanism. Conditioning on database presence (*C*)—which inevitably occurs in claims-based analyses—creates a collider structure (*A* → *C* ← *Y*) that opens biasing paths between exposure and outcome. The dotted line between *S*_1_ and *S*_2_ represents their correlation, further amplifying selection bias. Consequently, the observed associations may represent artifacts of the bias structure rather than true causal effects.

Common effects often have their own common causes. Employment status or education level may influence access to both dental and medical coverage (Fig. 4) (Markowitz et al. 1991; Yamada et al. 2014). These socioeconomic factors (L) frequently serve as common causes of both the selection mechanism and systemic outcome of interest. When external data are available on individuals not included in the analytic sample (such as patients with medical coverage but lacking dental coverage), these complex biases can be addressed through principled transportability methods (Degtiar and Rose 2023). The inverse probability of selection weighting (IPSW) creates a pseudo-population in which selection into the claims database is independent of potential outcomes by weighting each individual by the inverse probability of their selection status (Carry et al. 2021). Specifically, individuals in the claims database receive weights equal to 1/P(S = 1|Z), where S = 1 indicates inclusion in the database and Z represents covariates that predict selection. These weights effectively up-weight individuals who are underrepresented in the claims data relative to the target population and down-weight those who are overrepresented. When applied correctly, IPSW can address the spurious associations created by conditioning on the collider (database inclusion) while also controlling for confounding pathways only when the same variables that predict selection also confound the exposure–outcome relationship. Detailed tutorials for implementing IPSW, including approaches for combining IPSW with inverse probability weighting for treatment assignment, are available in the recent methodological literature (Cole and Hernán 2008; Carry et al. 2021).

## Distinguishing Selection Bias from Generalizability

While both selection bias and generalizability issues relate to study population selection, their causal implications differ fundamentally based on timing of conditioning relative to treatment (Lash et al. 2020). Selection bias occurs when researchers condition on variables measured after or concurrent with treatment assignment (i.e., variables that are affected by the periodontal intervention, systemic outcome, or their causes), thereby creating collider structures that open noncausal backdoor paths (Hernán and Monge 2023). This threatens the internal validity of causal estimates within the study population itself. Generalizability issues, in contrast, arise when conditioning on pretreatment variables affects study sample composition relative to the target population. These pretreatment selection mechanisms primarily affect external validity (the population to which results can be generalized) but may preserve internal validity of effect estimates within the studied population (Degtiar and Rose 2023).

For example, if a claims database includes only patients with private insurance (a pretreatment characteristic), this creates a generalizability issue: results may be internally valid for privately insured patients but may not generalize to publicly insured or uninsured populations. However, if researchers condition on variables such as “adherence to follow-up appointments” (which may be affected by treatment success), this creates selection bias that threatens internal validity. The distinction lies in whether the conditioning variable could be affected by the treatment or outcome under study and the temporal ordering of these relationships.

## When Advanced Causal Methods Are Necessary in Periodontal Research

Dental claims data present unique challenges that make traditional approaches particularly vulnerable to bias beyond those found in general epidemiological studies. The dual coverage requirement inherent in dental–medical claims linkage creates selection mechanisms that can systematically distort causal estimates. Unlike population-based cohort studies in which participants are often recruited regardless of insurance status, claims-based periodontal research is inherently restricted to individuals with both dental and medical insurance coverage. Recent methodological work has demonstrated that quasi-experimental approaches can help address confounding by indication in dental claims studies, sometimes revealing that previously reported benefits of dental interventions were overstated due to healthy-user bias (Taylor et al. 2024).

Several methodological challenges specific to dental research compound the bias issues inherent in claims data. First, the definition of dental treatments in claims databases often lacks the precision required for causal inference. Unlike well-defined pharmaceutical interventions, “periodontal therapy” can encompass a heterogeneous mix of scaling, root planing, and surgical procedures with varying protocols and intensities. In addition, CDT codes do not consistently reflect disease stage or severity, creating challenges for defining exposures appropriately. This imprecision violates the consistency assumption necessary for causal interpretation, as the same treatment code may represent fundamentally different interventions across providers and patients.

## Practical Implementation Tools and Software for Dental Claims Research

### DAG Construction and Analysis

Researchers can construct and analyze DAGs using freely available software. DAGitty (www.dagitty.net) provides a web-based interface for drawing causal graphs and testing backdoor criteria, while the R package “dagitty” enables programmatic DAG analysis and integration with statistical workflows (Textor et al. 2016). When building DAGs for dental claims studies, researchers should systematically consider (1) common causes of both exposure and outcome, (2) temporal ordering of all variables, and (3) selection mechanisms inherent in dual coverage requirements. In high-dimensional dental claims datasets, researchers should prioritize variables based on subject-matter knowledge, focusing on established risk factors for both periodontal disease and systemic outcomes (e.g., smoking, age, socioeconomic status) while avoiding the inclusion of intermediate variables that may be on the causal pathway.

### Software Implementations for G-Methods

The g-methods described by Naimi et al. (2017) can be implemented using several statistical packages. For the G-formula, researchers can use the “gformula” package in R or implement parametric approaches following established tutorials. Inverse probability weighting can be executed using the “ipw” and “WeightIt” packages in R, with the latter providing flexible propensity score estimation and diagnostic tools (Pourhoseingholi et al. 2012). For inverse probability of selection weighting specifically, the approach described by Cole and Hernán (2008) can be implemented by first estimating selection probabilities using logistic regression, then applying stabilized weights in subsequent analyses.

### Triangulating Evidence through Multiple Administrative Data Applications

Claims data can be particularly valuable when used to triangulate findings from traditional observational studies through multiple analytical approaches. Target trial emulation represents one such approach, involving explicit definition of the protocol of a hypothetical randomized trial and then emulating this trial using observational data (for detailed implementation guidance, see Hernán et al. 2022). This method addresses common biases in claims analyses including temporal ambiguity, immortal time bias, and improper patient selection while leveraging scale and real-world representativeness of administrative data. In addition, claims databases can support comparative effectiveness research, pharmacovigilance studies, and health services research that complements findings from traditional epidemiological studies (Blonde et al. 2018).

### Decision Framework for Method Selection

Researchers should consider several key questions when determining appropriate analytical approaches: (1) Is the research question causal or purely associational? (2) Are external data available on nonselected individuals for IPSW? (3) What is the complexity of confounding structure (simple confounders vs. time-varying with feedback)? (4) Are there practical constraints on data collection and analysis complexity? This systematic approach helps determine whether traditional epidemiological methods suffice or whether advanced causal inference techniques are necessary for valid inference in periodontal–systemic disease research.

### Checking Causal Inference Assumptions

The validity of causal estimates from observational claims data depends critically on the plausibility of underlying assumptions. For positivity, researchers should examine propensity score distributions for extremely low probabilities or very large weights suggesting practical violations (Martin et al. 2024). The consistency assumption requires substantive knowledge about treatment definition, examining whether treatment codes represent sufficiently homogeneous interventions. The exchangeability assumption remains most challenging to assess. Researchers can employ several strategies: conducting E-value sensitivity analyses to quantify how strong an unmeasured confounder would need to be to explain away observed associations, examining he balance of measured covariates after weighting as a proxy for unmeasured variable balance, and triangulating findings across different data sources and study designs (VanderWeele and Ding 2017). Sensitivity analyses are particularly important in dental claims research where unmeasured confounders such as oral hygiene behaviors, health-seeking attitudes, or genetic predispositions may influence both periodontal treatment decisions and systemic health outcomes.

### Systematic Patterns in Current Periodontal–Systemic Claims Research

Our critical review of 7 recent studies examining periodontal–systemic relationships using claims data reveals systematic methodological gaps in addressing selection bias, despite growing recognition of this issue in broader epidemiological literature. These studies, spanning multiple countries and health systems from 2019 to 2025, demonstrate remarkably consistent methodological patterns that highlight systematic challenges in translating causal inference theory into dental research practice. While analytical approaches vary in sophistication, all share a fundamental characteristic: extensive attention to confounding adjustment with systematic neglect of selection bias mechanisms inherent in dual coverage requirements.

### Predominant Focus on Confounding over Selection Bias

Across all reviewed studies, the predominant analytical strategy focused exclusively on confounding adjustment through regression, propensity score matching, or inverse probability weighting for treatment assignment. While these approaches address important sources of bias, they systematically ignore selection mechanisms inherent in claims-based research. This pattern reflects a broader disconnect between the analytical methods used and the data structure being analyzed.

### Limited Methodological Innovation

Despite sophisticated statistical methods employed in individual studies, none implemented analytical approaches specifically designed to address collider structures created by dual coverage requirements. Only 1 study explicitly acknowledged this selection mechanism, noting their population had “voluntarily purchased insurance for dental care,” creating a group with “higher health awareness,” yet did not implement corresponding analytical corrections (Beukers et al. 2023).

## Conclusion

This study of periodontal–systemic disease relationships using administrative health care data offers unprecedented opportunities for advancing dental knowledge but presents significant methodological challenges that must be addressed to ensure valid causal inference. As demonstrated in our analysis, selection bias introduced by conditioning on dual coverage status can not only distort effect estimates but also potentially reverse the apparent direction of causal relationships between periodontal interventions and systemic outcomes.

These concerns align with broader critiques of oral–systemic research methodology, in which experts have noted that despite most studies posing causal questions, methodological deficiencies limit their interpretability and potential for meaningful clinical application (Raittio and Farmer 2021). We advocate for greater methodological rigor in periodontal–systemic disease research through 3 specific recommendations: (1) routine implementation of DAGs using freely available software tools to explicitly model selection mechanisms as potential collider structures in claims-based research; (2) application of appropriate correction methods such as IPSW when data on nonselected individuals are available, implemented using established statistical packages; and (3) transparent reporting of the potential bias impact on effect estimates, including sensitivity analyses where feasible. By adopting these approaches, periodontal researchers can strengthen the evidence base for oral–systemic relationships and provide more reliable guidance for clinical decision-making. Only through such methodological improvements can oral–systemic research provide the robust evidence base needed to support evidence-based clinical recommendations and public health policies that ultimately benefit patients through more effective targeting of periodontal interventions for systemic disease prevention and management.

## Author Contributions

J.J. Wong, contributed to conception, protocol writing, and research design, conducted and directed analysis, produced the first draft of the manuscript, reviewed and provided input into the manuscript; A. Carrasco-Labra, contributed to conception, protocol writing, and research design, reviewed and provided input into the manuscript; E.F. Schisterman, contributed to conception, protocol writing, and research design, conducted and directed analysis, produced the first draft of the manuscript, reviewed and provided input into the manuscript; O. Urquhart, M. Glick, contributed to conception, protocol writing, and research design, conducted and directed analysis, reviewed and provided input into the manuscript. All authors gave final approval and agree to be accountable for all aspects of the work.

## Supplemental Material

sj-docx-1-jdr-10.1177_00220345251387660 – Supplemental material for Addressing Selection and Confounding Biases in Dental Claims Data: A Causal Inference Framework for Periodontal–Systemic Disease Research Supplemental material, sj-docx-1-jdr-10.1177_00220345251387660 for Addressing Selection and Confounding Biases in Dental Claims Data: A Causal Inference Framework for Periodontal–Systemic Disease Research by J.J. Wong, O. Urquhart, A. Carrasco-Labra, E.F. Schisterman and M. Glick in Journal of Dental Research
