# Supplementary material for: Addressing Selection and Confounding Biases in Dental Claims Data: A Causal Inference Framework for Periodontal–Systemic Disease Research
Source: J Dent Res. 2025 Nov 25;105(1):51–8. doi: 10.1177/00220345251387660 (PMC12701901; doi:10.1177/00220345251387660)
Supplement: sj-docx-1-jdr-10.1177_00220345251387660 – Supplemental material for Addressing Selection and Confounding Biases in Dental Claims Data: A Causal Inference Framework for Periodontal–Systemic Disease Research [file sj-docx-1-jdr-10.1177_00220345251387660.docx]

**Table. Study Design and Selection Bias Methodology in Claims-Based Periodontal-Systemic Disease Studies (2019-2025)**

| **Study** | **N** | **Country/Insurance System** | **Cohort Design & Coverage Requirements** | **Exposure Definition** | **Outcome** | **Selection Bias Structure** | **Selection Bias Acknowledged** | **Selection Bias Methods** | **Confounding Adjustment** |
| --- | --- | --- | --- | --- | --- | --- | --- | --- | --- |
| **Raedel et al. 2019** | 415,718 | Germany / BARMER (major German statutory health insurance fund, 8.5M members representing ~10% of German population; comprehensive national coverage including dental/medical integration) | Retrospective cohort 2012-2015; requires insurance membership for both dental and medical claims; controls sampled from 5.7M members without periodontal treatment; certain German regions excluded due to missing data | "Periodontal treatment" per German insurance: clinical evaluation, scaling/root planing, optional surgery, up to 4 re-evaluations; paid by insurance except pre-treatment hygiene | First tooth extraction at patient level; 60-day post-treatment exclusions | Dual coverage requirement: dental insurance for treatment documentation, medical insurance for extraction claims | Not acknowledged | None | Demographic matching (age 5-year blocks, gender, federal state) using weighted sampling with replacement |
| **Choi et al. 2021** | 748,792 | USA / Commercial insurer (unnamed major US commercial insurer with >60M beneficiaries; represents ~18% of commercially insured Americans with integrated medical-dental coverage) | Retrospective cohort 2015-2019; requires continuous enrollment ≥6 months before/after first pregnancy care; excludes non-continuously enrolled; analysis unit = individual pregnancies | "Periodontal disease" inferred from CDT procedure codes (16 specific codes listed); requires ≥4 distinct visits; assumes "treated=recovered" if completed before pregnancy, otherwise "has PD" | Adverse pregnancy outcomes: LBW (<2500g), PTB, spontaneous abortion via ICD codes | Dual coverage requirement: dental insurance for CDT codes, medical insurance for ICD pregnancy codes | Misunderstood ("avoid selection bias as subject population chosen effectively randomly from entire population of women userbase") | None | Logistic mixed-effects model with patient/zip code random effects; adjusted for age, 7 clinical conditions, geography (zip code as SES proxy) |
| **Blaschke et al. 2022** | 21,263 | Germany / InGef research database (≥6M Germans from ~70 statutory health insurers, predominantly company-based health insurance funds; represents employed population with comprehensive medical-dental integration) | Retrospective cohort 2011-2016; continuously insured; newly diagnosed CHD in 2013; exposed = ≥1 periodontal treatment in index quarter + 8 subsequent quarters; unexposed = no treatment in index quarter + following 2 years | "Periodontal treatment" via German BEMA fee codes P200-P203, 108, 111; no differentiation by number of treatments performed | Healthcare costs (total, inpatient, outpatient, drugs) measured in third year after CHD diagnosis | Dual coverage requirement: statutory health insurance for both dental treatment documentation and medical CHD diagnosis/costs | Not acknowledged | None | Doubly robust method (outcome regression + inverse probability weighting); age, sex, Charlson comorbidity index, healthcare utilization patterns, geographic region |
| **Beukers et al. 2023** | 1,224,457 (1,121,790 for multivariable) | Netherlands / Achmea insurance company database (major Dutch insurer covering >1M lives; represents ~6% of Dutch population with dual medical-dental coverage) | Longitudinal cohort 2007-2014; requires compulsory medical healthcare insurance PLUS voluntary dental care insurance; 8-year continuous coverage; excludes interrupted insurance periods; focuses on nonfatal ACVD only | "PD status" from dental care insurance claim codes; "any first appearing PD claim code sufficient to define PD status"; structured consecutive coding system required | Nonfatal ACVD events from medical care insurance claim codes; time-dependent survival analysis | Dual coverage requirement: compulsory medical insurance + voluntary dental insurance purchase | Explicitly acknowledged ("higher health awareness," "voluntarily purchased insurance for dental care") | None applied despite acknowledgment | Time-dependent Cox models with DAG guidance + E-values; age, sex, socioeconomic position (postal code proxy), diabetes mellitus |
| **Michalowicz et al. 2023** | 9,503 (4,057 CAD; 3,247 CBVD; 4,879 T2D) | USA / HealthPartners (integrated healthcare system providing both care and insurance to >1M members across Minnesota and Wisconsin; represents mixed commercial/Medicare population in upper Midwest with comprehensive medical-pharmacy-dental integration) | Retrospective cohort 2004-2017; requires continuous medical, pharmacy, AND dental insurance coverage ≥1 year before and after concurrent disease diagnoses; integrated clinic records and claims data | "Periodontal treatment" via CDT codes (D4341, D4342, D4355, D4210, D4211, D4240, D4241, D4245, D4260, D4261, D4263-7, D4264, D4274, D4910); categorized as active therapy alone, active+maintenance, maintenance only, or no care | Hospitalizations, medical costs (total, inpatient, outpatient, pharmacy), glycated hemoglobin, cardiovascular events, death | Triple coverage requirement: continuous medical + pharmacy + dental insurance; creates informative censoring when coverage discontinues | Not acknowledged | None | Time-dependent stabilized inverse probability weights (IPW) for treatment and censoring; LASSO variable selection; extensive covariates including demographics, comorbidities, periodontal severity, insurance type |
| **Thakkar-Samtani et al. 2023** | 671,483 (390,783 Medicaid; 280,700 commercial) | USA / IBM MarketScan Commercial Insurance and Medicaid databases (13 deidentified states for Medicaid representing diverse geographic regions; select commercial plans across all 50 states covering ~15M commercially insured lives; captures socioeconomically diverse diabetes population across public/private insurance divide) | Retrospective cohort 2013-2019; adults aged 21-64 continuously enrolled 2017-2019; initial DM diagnosis 2013-2018, continued DM in 2019; excludes patients ≥65 due to "Medicaid and Medicare dual coverage" | "Periodontal treatment" via CDT codes D4000-D4999; treatment = ≥1 periodontal visit 2017-2018; control = no periodontal treatment visit | Overall healthcare costs (outpatient, inpatient, drug) in 2019; cost outcomes measured 1-2 years after treatment period | Dual coverage requirement: continuous enrollment in medical and dental insurance; acknowledges coverage exclusions for elderly due to "dual coverage" complexity | Not acknowledged (aware of coverage complexity but not selection bias implications) | None | Propensity score matching (PSM) + generalized linear modeling with gamma distribution; age, sex, race, past preventive visits, past medical costs, Elixhauser comorbidity score |
| **Sakamoto et al. 2025** | 4,010 | Japan / JMDC (Japan Medical Data Center aggregating claims from multiple health insurance societies covering ~17M people as of April 2024; represents diverse Japanese employed population across company-based health insurance systems with integrated medical-dental claims) | Retrospective cohort 2012-2020; requires both medical claims (diabetes diagnosis E11/E14 + medication ATC A-10) AND dental claims (periodontal management codes); continuous periodontal management for 2 years post-diabetes onset required; age ≥30 years; excludes those without lifestyle/HbA1c data | "Periodontal disease management" via 23 specific practice codes (304000410, 304000510, 304000610, etc.) performed ≥1 time for 2 consecutive years before diabetes onset | Medical costs associated with diabetes mellitus (ICD10 E10-14); HbA1c levels | Dual coverage requirement: dental insurance for periodontal treatment documentation, medical insurance for diabetes diagnosis/medication; creates classic A→C←Y collider | Partially acknowledged but misunderstood - mentions database limitations (age/job restrictions, excludes ≥75 years, those without insurance) but does NOT acknowledge dual coverage requirement creates systematic selection bias | None | Basic statistical methods only - Mann-Whitney U tests, χ² tests, stratified analyses by gender; no sophisticated causal inference methods (no propensity scores, regression adjustment, matching, or multivariate analyses) |

**Table 1.** Systematic analysis of selection bias methodology in claims-based periodontal-systemic disease studies (2019-2025). Seven studies encompassing 3,095,226 patients across four countries (Germany, United States, Netherlands, Japan) and diverse healthcare financing models were systematically reviewed. Studies represented insurance systems, ranging from statutory/single-payer models (3/7 studies) to private/commercial-dominant systems (4/7 studies), demonstrating significant socioeconomic diversity including Medicaid beneficiaries, commercially insured populations, and universal coverage systems. **Coverage Requirements:** All studies required dual (6/7 studies) or triple (1/7 studies) insurance coverage for inclusion, with no studies including patients with single coverage requirements. **Selection Bias Recognition:** Selection bias from dual/triple coverage requirements was not acknowledged in 4/7 studies, explicitly misunderstood in 1/7 studies, partially acknowledged but misunderstood in 1/7 studies, and acknowledged but uncorrected in 1/7 studies. Notably, 0/7 studies implemented statistical methods specifically designed to address selection bias. **Confounding Control:** Advanced causal inference methods were employed in 6/7 studies (propensity score matching, inverse probability weighting, doubly robust methods, directed acyclic graphs with E-values), while basic statistical methods only were used in 1/7 studies. **Major Methodological Pattern:** 6/7 studies applied sophisticated causal inference methods for confounding control while 0/7 studies implemented selection bias correction techniques. This persisted across all countries and healthcare systems examined, spanning six years of research and affecting over 3 million patients, suggesting fundamental conceptual oversight rather than system-specific limitations.
